# Effect of contact lens material on cytotoxicity potential of multipurpose solutions using human corneal epithelial cells

**Published:** 2011-12-28

**Authors:** M.B. Gorbet, N.C. Tanti, B. Crockett, L. Mansour, L. Jones

**Affiliations:** 1 Department of Systems Design Engineering, University of Waterloo, Waterloo, Ontario, Canada; 2 Centre for Contact Lens Research, School of Optometry, University of Waterloo, Waterloo, Ontario, Canada; 3 Biomedical Sciences, University of Waterloo, Waterloo, Ontario, Canada

## Abstract

**Purpose:**

Multipurpose solutions (MPS) are used daily to clean and disinfect silicone hydrogel (SiHy) contact lenses. This in vitro study was undertaken to identify the potential for interaction between MPS, SiHy surface treatments, and lens materials, which may lead to changes in the response of human corneal epithelial cells (HCEC) to MPS-soaked lenses.

**Methods:**

The MPS tested were renu fresh (formerly known as ReNu MultiPlus; ReNu), OptiFree Express (OFX), OptiFree RepleniSH, SoloCare Aqua, and Complete Moisture Plus. The SiHy materials evaluated were lotrafilcon A, lotrafilcon B, comfilcon A, galyfilcon A, and balafilcon A (BA). MPS-soaked lenses were placed on top of adherent HCEC. The effect of MPS dilutions (0.1 to 10% final concentration in medium) was also characterized. Cell viability, adhesion phenotype and caspase activation were studied after 24-h cell exposure. OFX released from lenses was determined using UV absorbance.

**Results:**

A significant reduction in viability (between 30 to 50%) was observed with cells exposed to lenses soaked in ReNu and OFX. A significant downregulation of α_3_ and β_1_ integrins, with integrin expression ranging from 60% to 75% of control (cells with no lens), was also observed with OFX and ReNu-soaked lenses. With the exception of BA, all other lenses soaked in OFX resulted in significant caspase activation, whereby over 18% of cells stained positive for caspases. Minimal caspase activation was observed in cells exposed to ReNu and Solo soaked lenses. For both OFX and ReNu, exposing cells to at least a 5% dilution had a significant effect on viability and integrin expression. While Complete and Solo did not lead to reduction in viability, cells exposed to a 10% dilution showed reduced integrin expression down to less than 70% of control value. Comparing cell response to diluted MPS solutions and various MPS-soaked lenses showed that it is not possible to reliably use cell response to MPS dilution alone to assess MPS biocompatibility.

**Conclusions:**

Our results demonstrate that the reaction of HCEC to MPS are affected by the type of lenses the MPS is released from and may potentially be influenced by the surface treatment (or lack of it) of SiHy materials.

## Introduction

Following removal from the eye, contact lenses must be placed in a contact lens solution for disinfection and to remove tear film deposits. The most commonly prescribed care systems are multi-purpose solutions (MPS), which are single solutions that are used to rinse, clean and disinfect contact lenses, and contain many different components to enhance disinfection and cleaning properties [1,2]. The disinfecting properties of MPS are conferred by the active biocide, which are commonly a polyquaternium, biguanide, or hydrogen peroxide agent [2,3]. The biocides in MPS, such as Polyquad®, Aldox®, and polyhexamethylene biguanide (PHMB), are intended to breach cell walls of microbes [4], but may also have the potential to cause corneal epithelial cell membrane toxicity [5-8]. MPS also contain a buffering solution to maintain the pH of the solution, which is typically either borate or phosphate-based [2]. The solution must be efficacious enough against microbial flora, but gentle enough to not cause adverse effects on the corneal surface, as the corneal surface will be exposed to some of the solution following lens insertion and this will remain in contact with the epithelium until washed away by the post-lens tear film [2,3,9]. Despite the high molecular weight of the disinfecting agents, there is now evidence that they can potentially adsorb or form a complex with other components of MPS on the lens [10-12], and can then be released onto the corneal surface post-insertion.

Silicone hydrogel (SiHy) lenses incorporate siloxane moieties to increase oxygen delivery to the cornea [13]. Various surface modifications and proprietary chemistry are employed to enhance the wettability of the SiHy lens surface and Table 1 lists the major features of several SiHy materials. The surface of lotrafilcon A (LA) and lotrafilcon B (LB) lenses are permanently modified with a mixture of trimethyl-silane oxygen and methane, in a gas plasma reactive chamber [14,15]. The resulting coating is a continuous ultra-thin, hydrophilic surface. Balafilcon A (BA) lenses are treated in a gas plasma chamber to convert all siloxane components into silicate compounds, making the surface more hydrophilic [1,13]. The transformed areas form ‘glassy islands’, which bridge over the underlying lens material [15,16]. This differs from the surfaces of most other SiHy lenses, as it is the only SiHy lens with a relatively “rough,” discontinuous surface. Galyfilcon A (GA) and comfilcon A (CA) are both non-surface treated lenses. In GA lenses, an internal wetting agent, derived from poly(vinylpyrrolidone), is incorporated into the bulk material to improve hydrophilicity [1,13]. To date, there is very little published on the surface characteristics of CA lenses. Proprietary chemistry is used to create a highly wettable surface, without specific surface modifications. The surface features are comparable to that of conventional polyHEMA lenses [15].

**Table 1:** Properties of lenses used in the study [27].

**Proprietary name**	**PureVision**	**Focus Night & Day**	**O_2_ Optix**	**Biofinity**	**Acuvue Advance**
Manufacturer	Bausch & Lomb	CIBA Vision	CIBA Vision	CooperVision	Vistakon (Johnson & Johnson)
USAN	Balafilcon A (BA)	Lotrafilcon A (LA)	Lotrafilcon B (LB)	Comfilcon A (CA)	Galyfilcon A (GA)
Water Content (%)	36	24	33	48	47
Dk	99	140	110	128	60
Charge	Ionic	Non-ionic	Non-ionic	Non-ionic	Non-ionic
Principle Monomers	NVP + TPVC	DMA + TRIS + siloxane macromere	DMA + TRIS + siloxane macromer	Undisclosed	mPDMS + DMA + EGDMA + HEMA + siloxane macromer + PVP
Surface Treatment	Plasma oxidation process	25 nm plasma coating	25 nm plasma coating	None	None, internal wetting agent

DMA (*N*,*N*-dimethylacrylamide); EGDMA (ethyleneglycol dimethacrylate); HEMA (poly-2-hydroxyethyl methacrylate); mPDMS (monofunctional polydimethylsiloxane); NVP (*N*-vinyl pyrrolidone); PVP (polyvinyl pyrrolidone) TPVC (tris-(trimethylsiloxysilyl) propylvinyl carbamate; TRIS (trimethylsiloxy silane).

Despite modifications aimed to reduce adsorption and release of active biocides onto the corneal surface, certain combinations of MPS and SiHy lenses can lead to a cytotoxic effect in vitro [17,18] and some combinations have the potential to exhibit excessive corneal staining in vivo [19-22]. Many biocompatibility studies have been undertaken to examine the effect of ophthalmic solutions, including contact lens packaging solutions and multipurpose solutions, on corneal and conjunctival cells [6,23-26]. Most in vitro studies with MPS have been limited to studying the effect of solutions on cells, using extracts or dilutions of solutions to evaluate the effect. There is evidence that exposure to MPS can cause cell death in vitro, either through apoptosis or necrosis [6,23,27,28]. Apoptosis is a programmed form of cell death and serves, among others, as a defense mechanism in the removal of damaged cells [29,30]. Apoptotic signaling pathways involve cysteine aspartate proteases (also known as caspases) as mediators for initiating cellular disassembly [29]. In comparison, necrosis is considered to be accidental or inappropriate, and occurs under extremely unfavorable conditions. It is an uncontrollable, irreversible form of cell death and it has not been determined whether signaling pathways mediate necrotic cell death [30]. Previous studies with MPS were able to evaluate the potential cytotoxic effect of ophthalmic and multipurpose solutions in vitro, and while this is valuable research, there is currently no information on the effect of the direct release of solutions from silicone hydrogel lenses on human corneal epithelial cell (HCEC).

This study was undertaken to determine the effect that the properties of SiHy contact lenses have on the cytotoxic potential of direct release of multipurpose solutions from various SiHy lenses, and to gain further understanding of the interactions between MPS solution, surface treatment, lens material and HCECs. Cell viability was assessed following exposure to MPS-soaked lenses, but the state of adherent cells was also investigated. Flow cytometry was used to determine how MPS release from contact lens may affect cell integrity, cell adhesion phenotype (by measuring levels of integrin expression) and cell apoptosis (by measuring caspase activation).

## Methods

### Reagents and antibodies

Keratinocyte serum free medium, growth supplement (Bovine Pituitary extract), and pen-strep solution were purchased from ScienCell (Carlsbad, CA). All other cell culture reagents, Dulbecco’s minimum essential medium, fetal bovine serum, phosphate buffer saline, and TriplExpress were purchased from Invitrogen (Burlington, Ontario, Canada). A sterile solution of Unisol 4® (BBS), an unpreserved borate-buffered saline (Alcon, Forth Worth, TX) was purchased from a commercial source and used within its expiration date.

Monoclonal antibodies to β_1_ integrin (CD29; Immunotech-Coulter, Marseilles, France) was fluorescein isothiocyanate (FITC) conjugates. The monoclonal antibody against α_3_ integrin (CD49c; Serotec, Mississauga, Ontario, Canada) was a R-phycoerythrin (PE) conjugate. Parafomaldehyde was purchased from Fisher Scientific (Ottawa, Ontario, Canada) and all other chemicals used to prepare Hepes Tyrode Buffer were of analytical or reagent grade.

### Contact lenses and multipurpose solutions

Six silicone hydrogel lens materials were tested (Table 1): balafilcon A (BA; Bausch & Lomb, Rochester, NY), lotrafilcon A (LA; CIBA Vision, Duluth, GA), lotrafilcon B (LB; CIBA Vision), comfilcon A (CA; CooperVision, Fairport, NY), and galyfilcon (GA; Vistakon, Jacksonville, FL). All lenses were purchased in their original packaging, had a diameter between 14.0 and 14.2 mm and a curvature of 8.5 to 8.7mm. Five polyquaternium or biguanide preserved multipurpose solutions were tested (Table 2).

**Table 2:** Disclosed composition of the MPS used in the study [28].

**Manufacturer**	**Brand (abbreviation)**	**Disinfecting Agent**	**Buffer**	**Other reported agents (surfactants and chelating agents)**
Alcon	Opti-Free Express (OFX)	Polyquad® 0.001%, Aldox® 0.0005%	Borate	Sorbitol; citrate (citric acid), 0.05% EDTA; poloxamine (Tetronic 1304), aminomethylpropanol (AMP-95)
Alcon	Opti-Free RepleniSH (OFR)	Polyquad® 0.001%, Aldox® 0.0005%	Borate	Citrate, poloxamine (Tetronic 1304), non-anoyl ethylene-diaminetriacetic acid, propylene glycol
AMO	Complete Moisture Plus (Complete)	PHMB 0.0001%	Phosphate	Taurine; 0.01% EDTA; Poloxamer 237 (Pluronic F87); HPMC 0.15%; propylene glycol
Bausch & Lomb	renu fresh (formerly ReNu MultiPlus; ReNu)	PHMB 0.0001%	Borate	Sodium borate; Hydroxyalkylphosphonate (Hydranate™); 0.1% EDTA; Poloxamine (Tetronic 1107)
CIBA Vision	SoloCare Aqua (Solo)	PHMB 0.0001%	Tris	Sorbitol; 0.025% EDTA; dexpanthenol (provitamin B5); Pluronic F127 (poloxamer 407)

PHMB: polyhexamethylene biguanide (also known as polyhexanide, Dymed, polyhexadine, and polyaminopropyl biguanide).

### In vitro cell culture

#### Immortalized human corneal epithelial cells (HCEC)

SV40-immortalized human corneal epithelial cells were cultured in keratinocyte serum free medium supplemented with bovine pituitary extract, recombinant epidermal growth factor and pen-strep (KSFM). Fresh medium was added every other day and cells were grown to 90% confluency in tissue culture treated flasks. Adherent cells were removed using a dissociation solution, TriplExpress (Sigma-Aldrich, Oakville, Ontario, Canada). Cells were routinely observed for any morphological changes.

#### In vitro model

A direct contact in vitro model was used [31]. Briefly, HCEC were seeded onto a 24 well tissue culture treated polystyrene (TCPS) plate at 10^5^ cells per well. Cells were left to adhere for 18–24 h in a humid CO_2_ incubator, which resulted in the formation of a monolayer of HCEC. Simultaneously, SiHy lenses were totally immersed in the MPS, in a sterile 12-well polystyrene plate and soaked for 18–24 h. Controls included lenses soaked in PBS. All lens-solution soaking combinations were performed under sterile conditions.

The next day after seeding, supernatant was removed and fresh serum-free medium was added. MPS-soaked SiHy lenses were placed gently on top of the monolayer, face-down, with the concave surface facing upwards and incubated for 24 h at 37 °C (5% CO_2_ in a humid incubator). Lenses were totally immersed in medium. After 24 h, lenses were carefully removed from wells. The lenses did not adhere to the HCEC monolayer. Lenses were also routinely observed for the presence of adherent cells on their surface and no HCEC proliferation on the lens was observed.

Additionally, cells were exposed directly to the MPS: MPS was added to the medium with a final concentration ranging from 0.1 to 10%. Cells were incubated for 24 h and cells were assessed for viability and activation.

#### Cellular viability

To assess cytotoxicity of the products released from the contact lenses, the 3-(4,5-dimethylthiazol-2-yl)-2,5-diphenyltetrazolium bromide (MTT) cellular viability assay was performed; the assay gives an indication of metabolically active cells and thus cell death or a reduction in metabolism can be measured relative to control samples. After a gentle rinse in sterile PBS, cells were incubated with a solution of MTT (at 1 mg/ml in KSFM medium; Calbiochem, La Jolla, CA). After 3 h at 37 °C, cells were lysed with DMSO and absorbance read at 595 nm (Thermo MultiSkan Spectrum Photometer, Fischer Scientific, Ottawa, ON, Canada). All results are expressed as relative viability compared to cells grown in the absence of a contact lens.

### Integrin expression

To determine if MPS release from the contact lens materials led to a change in cell adhesion phenotype, levels of integrin expression were determined on cells that were still adherent following incubation with MPS-soaked lenses. HCEC were removed from the wells with TrypLExpress (Invitrogen), following a gentle wash in PBS. Cells were washed and resuspended in DMEM/FBS. Small aliquots (30 μl) of HCEC, suspended in DMEM-FBS, were incubated with saturating concentration of fluorescently-labeled antibodies for 1 h at 4 °C. Samples were then diluted in Hepes Tyrode Buffer, fixed in 1% paraformaldehyde (final concentration) and analyzed by flow cytometry within 5 days.

### Caspase activation

To determine if exposure to MPS release led to cell apoptosis, caspase activation on adherent cells was studied. HCEC were removed from the wells with TrypLExpress (Invitrogen), following a gentle wash in PBS. Cells were washed and resuspended in DMEM/FBS.

Small aliquots of HCEC, diluted in DMEM/FBS, were incubated with a fluorescently-labeled pan caspase inhibitor (FITC-VAD-FMK; Calbiochem, San Diego, CA) for 1 h at 37 °C. Samples were washed and resuspended in wash buffer, before immediate analysis by flow cytometry.

### Flow cytometry acquisition/analysis

All integrin, annexin V, and caspase samples were acquired on a Becton Dickinson FACSVantage flow cytometer (Mountain View, CA) using CELLQuest Software. Appropriate isotype controls were used with each experiment. Analysis was also performed using FACSExpress post data acquisition.

### Lens release profile

The release of Opti-Free Express (OFX) from contact lenses was characterized by UV absorbance. OFX – soaked lenses were incubated in 700 µl PBS for 24 h at 37 °C. Lenses were removed and the lens extracts were stored in glass vials at 4 °C until analysis. The day of the analysis, extracts were transferred to thoroughly cleaned quartz cuvettes, and absorbance was read on a UV spectrophotometer (Thermo MultiSkan Spectrum Photometer; Fischer Scientific, Ottawa, ON, Canada) in the range of 200 – 250 nm. For each experiment, serial dilutions of OFX solution in PBS were used for the calibration curve. Absorbances of PBS alone were also measured to determine background absorbance.

### Statistical analysis

All results are reported as means±standard deviation (SD). To evaluate the significance of the differences in cell viability and cell activation, an ANOVA was performed, followed by multiple pair-wise comparisons using the Tukey HSD test using Statistica V8 (StatSoft, Tulsa, OK). Samples were compared to PBS soaked lenses, as well as cells grown in the absence of a contact lens. Significant differences between MPS solutions are also reported. A p value of <0.05 was required for statistical significance. The number of experiments was equal to or greater than three with different cell passages. For each experiment, all solutions were tested at the same time.

## Results

### Cell viability and integrin expression with MPS-soaked lenses

**Figure 1:**
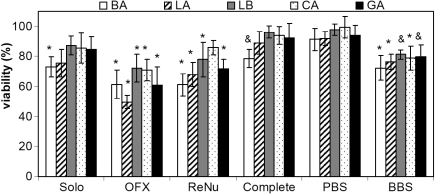
HCEC viability after 24 h contact with Lotrafilcon A (LA), Balafilcon A (BA), Lotrafilcon B (LB), Galyfilcon A (GA), and Comfilcon A (CA) lenses soaked in various MPS. Viability was measured by MTT assay and is expressed as a percentage relative to cells grown in the absence of lenses. n=4 to 5, ^*^ significantly different from cells grown in the absence of lens and PBS-soaked lens (p<0.045). ^&^ significantly different from cells grown in the absence of lens only (p≤0.01). Complete - Complete Moisture Plus; OFX - Opti-Free Express; ReNu - ReNu MultiPlus; Solo - SoloCare Aqua.

When testing combinations of lens-solutions, there was no significant difference between cells exposed to PBS-soaked lenses and cells cultured in the absence of a lens, indicating that the presence of a lens itself did not reduce cell viability and thus differences in viability were induced by the products released from the lens. As shown in Figure 1, for all types of lenses soaked in OFX, a significant reduction in viability was observed (p<0.002). With OFX-soaked lenses, LA also had significantly lower viability compared to BA, LB, and CA lenses (p<0.01). With lenses soaked in ReNu and Solo, LA and BA, as well as GA (soaked in ReNu only) also significantly reduced cell viability.

**Figure 2:**
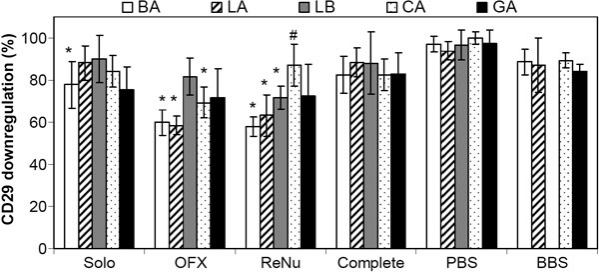
HCEC β_1_ (integrin CD29) expression after 24 h contact with Lotrafilcon A (LA), Balafilcon A (BA), Lotrafilcon B (LB), Galyfilcon A (GA), and Comfilcon A (CA) lenses soaked in various MPS. Integrin expression was measured by flow cytometry and is expressed as a percentage relative to cells grown in the absence of a lens. n=3 to 4, ^*^ significantly different from cells grown in the absence of lens and PBS-soaked lens (p<0.045), ^#^ significantly different from respective buffer control soaked lens (p≤0.04). Complete - Complete Moisture Plus; OFX - Opti-Free Express; ReNu - ReNu MultiPlus; Solo - SoloCare Aqua; BBS - borate buffer saline; PBS - phosphate buffer saline.

**Figure 3:**
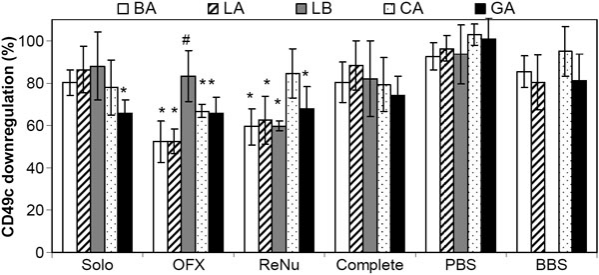
HCEC α_3_ (integrin CD49c) expression after 24 h contact with Lotrafilcon A (LA), Balafilcon A (BA), Lotrafilcon B (LB), Galyfilcon A (GA), and Comfilcon A (CA) lenses soaked in various MPS. Integrin expression was measured by flow cytometry and is expressed as a percentage relative to cells grown in the absence of a lens. n=4 to 5, ^*^ significantly different from cells grown in the absence of lens and PBS-soaked lens (p<0.04), ^#^ significantly different from its respective buffer control soaked lens (p≤0.04). Complete - Complete Moisture Plus; OFX - Opti-Free Express; ReNu - ReNu MultiPlus; Solo -SoloCare Aqua; BBS - borate buffer saline; PBS - phosphate buffer saline.

Upon 24-h contact with soaked lenses, a downregulation of integrin expression was observed. As shown in Figure 2 and Figure 3, depending on the lens tested, OFX and ReNu soaked lenses led to a significant reduction (between 22 and 40%) in β_1_ and α_3_ expression. As both OFX and ReNu are borate-buffered based solution, lenses were also soaked in borate buffer (BBS). While there was a 10 to 20% reduction in β_1_ and α_3_ with BBS-soaked lenses, it was not significant compared to PBS-soaked lenses. These results suggested that the significant downregulation observed with OFX and ReNu lens combinations (OFX-soaked LA, BA, and CA lenses and ReNu-soaked LA, BA, and LB lenses) was due to interactions between the lens and active components of the cleaning solutions and not the borate buffer.

### Caspase activation

**Figure 4:**
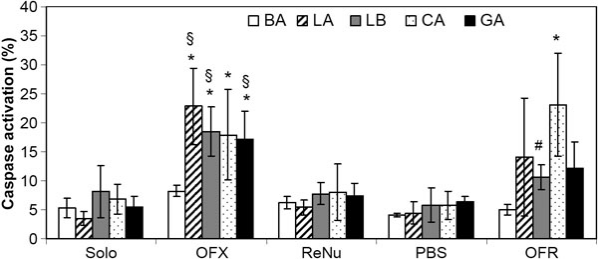
HCEC caspase activation after 24 h contact with Lotrafilcon A (LA), Balafilcon A (BA), Lotrafilcon B (LB), Galyfilcon A (GA), and Comfilcon A (CA) lenses soaked in various MPS. Activation was measured by flow cytometry and the percentage of cells staining positive for caspase activation is reported. Apoptosis induced by 24 h growth factor withdrawal or exposure to 10% alcohol resulted in 46±15% and 26±13%, respectively, of cells staining positive for caspase activation. n=3 to 4, ^*^ significantly different from cells grown in the absence of lenses (p<0.0006), ^§^ significantly different from its respective PBS, Solo, ReNu soaked lens (p<0.01), ^#^ significantly different from respective OFX-soaked lens (p<0.005). OFX - Opti-Free Express; OFR - Opti-Free RepleniSH; ReNu - ReNu MultiPlus; Solo - SoloCare Aqua; PBS - phosphate buffer saline.

Activated caspases were detected by flow cytometry: the fluorescently-tagged pan caspase inhibitor (FITC-VAD-FMK) fluoresces most intensely in cells with active caspases. Significant caspase activation in cells, which occurred in up to 25% of cells stained for capsase, was observed at 24 h with some lenses soaked in OFX (Figure 4). BA-OFX did not induce caspase activation and was significantly different from all other OFX soaked lenses (p<0.05). Testing a 10% dilution of OFX resulted in caspase activation that was similar to control cells (10% OFX: 7±1%; control cells 6±2%). Since OFX and OFR used the same biocides, lenses soaked in OFR were also tested, and significant caspase activation (albeit reduced compared to OFX) was also observed with OFR-soaked lenses. Neither lenses soaked in BBS nor in ReNu induced caspase activation. With the exception of CA lenses, lenses soaked in OFR appeared to induce less caspase activation compared to OFX. A significant effect of lens type on caspase activation (p=0.000005) and a significant interactive effect with solution type (p=0.00007) were also found. The increase in caspase activation with OFX-LA lenses was also verified by assessing the level of proteolytic enzyme activity of caspase 3 (C3): OFX-LA lenses led to a C3 activity of 24±10 AU (arbitrary unit), compared to 10±3 AU for control cells (no lens, PBS, or BBS soaked LA).

### Cell viability and integrin expression with diluted MPS

When testing MPS dilutions, a significant effect of concentration was observed for OFX and ReNu on cell viability (Table 3, first column). OFX (10%) and 10% ReNu were found to be significantly different from 1% of their own solutions (p<0.002). A linear relationship could be observed between concentration and viability for OFX and ReNu. This finding was in contrast to Complete and SoloCare, which contain the same biocide or disinfecting agent as ReNu, but different buffering solutions: Complete and SoloCare did not adversely affect viability with increasing concentrations, with both solutions exhibiting over 80% viability. PBS and BBS were also tested as controls, and a significant effect of concentration was observed with BBS but not PBS, suggesting that the buffering agent may be in some part responsible for the observed reduced viability observed with OFX and ReNu.

**Table 3:** Effect of MPS concentration on cell viability, β_1_ (integrin CD29) and α_3_ (integrin CD49c) expression after 24 h incubation.

**MPS**	**Final concentration**	**Cell viability (%)**	**β1 expression (%)**	**α3 expression (%)**
Solo	10%	79.5±10.2	58.9±5.2^*^	59.5±13.1^*^
	5%	90.0±9.7	80.0±1.0	72.8±11.0
	1%	91.4±11.0	95.5±1.7	87.0±2.4
OFX	10%	67.0±9.7^#^	55.5±6.6^*^	63.0±7.3^*^
	5%	73.5±6.7^*^	53.2±3.6^*^	55.3±5.7^*^
	2%	87.9±3.1	81.9±6.1	73.0±5.0
	1%	87.0±5.3	96.4±9.5	83.8±9.3
ReNu	10%	63.0±7.4^#^	49.9±4.9^*^	59.4±6.7^*^
	5%	74.6±6.9^*^	57.0±6.0^*^	58.3±4.7^*^
	1%	89.7±8.9	94.0±6.3	99.5±1.2
Complete	10%	86.0±6.5	68±2.7^*^	72.8±6.6^*^
	5%	95.0±4.5	90.7±3.8	90.7±2.3
	1%	102±9.3	99.0±7.5	96.8±8.3
PBS	10%	97.7±6.6	96.9±3.6	91.0±11.9
	5%	93.4±9.9	98.9±1.3	84.8±3.7
	1%	98.0±4.8	95.3±4.6	83.7±4.7
BBS	10%	68.5±8.1^#^	91.0±3.6	83.5±4.8
	5%	81.2±3.6	99.2±1.2	99.5±9.6
	1%	95.6±7.6	101±3	88.9±15

Viability was measured by MTT assay and is expressed as a percentage relative to cells grown in the absence of lenses, n=4 to 5, mean±standard deviation. Integrin expression was measured by flow cytometry and is expressed as a percentage relative to cells grown in the absence of a lens, n=3 to 4, mean±standard deviation. ^#^ Significantly different from cells exposed to PBS and 1% dilution of respective solution (p<0.002), ^*^ Significantly different from no solution and PBS (p<0.04).

As shown in Table 3, exposure to diluted MPS also led to reduced levels of integrin expression. Compared to the viability results, where there was a significant effect of concentration only with OFX and ReNu, MPS concentration had a significant effect on integrin expression for all solutions tested. For the 10% dilution, all solutions showed a 30 to 50% reduction in β_1_ and α_3_ expression.

### OFX release from soaked lenses

**Figure 5:**
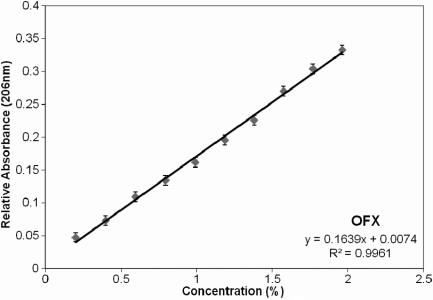
Calibration curve of absorbance of OFX, at 206 nm. Regression analysis yielded a trendline and formula and was used to approximate OFX concentration released from contact lenses, given the absorbance at 206 nm. OFX - Opti-Free Express.

**Figure 6:**
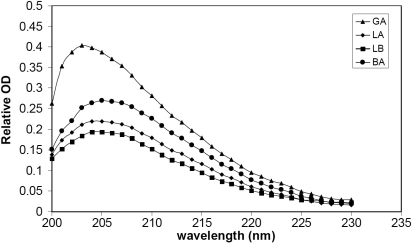
Absorption spectra for OFX soaked Lotrafilcon A (LA), Balafilcon A (BA), Lotrafilcon B (LB), Galyfilcon A (GA), and Comfilcon A (CA) extracts. Lenses were soaked overnight in OFX and lens release was performed in PBS for 24 h at 37 °C, 5% CO_2_. OFX - Opti-Free Express.

To determine the potential concentration that cells were exposed to following incubation with MPS-soaked lenses, further experiments were performed with OFX-soaked lenses, as its presence in solution can readily be identified by UV spectrophotometry. Absorbance scans (200 nm to 240 nm) of serial dilutions of OFX (2% to 0.2% in PBS) were performed. A peak in absorbance was typically observed between 202 and 206 nm. Based on the absorbance value at 206 nm (A_206_) of each concentration, a linear standard curve of A_206_ versus OFX concentration was obtained (Figure 5); A_206_ was chosen as it consistently provided the best linear standard curves when compared to the one obtained with values for A_202_, A_203_, A_204_, or A_205_. This curve was used to determine OFX release from lenses and UV absorbance of lens extracts, obtained following a 24 h incubation of OFX-soaked lenses in PBS, were measured (Figure 6). Using the standard curve and absorbance value of the lens extracts at 206nm, the concentration of OFX present in the extract was obtained. For all OFX-soaked lenses, the release determined by UV absorbance was found to be less than 3% (Table 4).

**Table 4:** Final OFX concentration in solution (%) released from lenses as measured by UV spectrophotometry at 206 nm.

**BA**	**LA**	**LB**	**CA**	**GA**
2.04±0.61	1.45±0.37	1.01±0.10	1.62±0.21	1.81±0.11

Lenses were soaked overnight in OFX and then incubated at 37 °C in PBS for 24 h. Released OFX concentration was calculated using absorbance of OFX standard dilutions in PBS at 206 nm.

## Discussion

The in vitro contact lens “onlay” model reported in this manuscript appears to be a valuable tool to study the effect of direct release of multipurpose solutions on corneal epithelial cells. No significant difference between PBS-soaked lenses and cells grown in the absence of a lens demonstrated that the presence of the lens itself did not induce mechanical damage. Monolayer epithelial cell cultures have been described as potentially over sensitive to MPS exposure [32]. However, recent studies on benzalkonium chloride toxicity have demonstrated similar cytotoxicity results using both a 3-D reconstituted model corneal epithelium and a monolayer of corneal epithelial cells [33,34], justifying our choice on using a monolayer to gain a better understanding on the mechanisms of lens-solution incompatibilities.

Due to the chemistry of the lens and its surface treatment, MPS uptake and release will differ between lenses [10,12] and the mechanisms of cytotoxicity of MPS may thus differ significantly between lenses. Our results provide evidence to support this hypothesis: significant effects were observed for cells exposed to diluted MPS, but not all MPS-lens combinations affected cells in the same way. From our OFX-soaked lens release study, it also appears that for most lenses, cells would be exposed to a maximum concentration of 2%. Exposing cells to 2% OFX over 24 h did not lead to significant change in viability and integrin expression, while all lens-OFX combinations did. This further highlights the role that lens uptake and release plays on solution biocompatibility. While blinking and the constant regeneration of the tear film may dilute the effects of the biocides in vivo, the uptake and slow release by the lens may also increase the exposure time to the cornea, which further supports the importance of evaluating solution release from a contact lens in vitro.

Integrins β_1_ and α_3_, which have strong roles in epithelial cell adhesion, were chosen as a means to assess the state of adherent cells and determine if incubation with MPS-soaked lenses led to a compromised corneal monolayer in vitro. α_3_, which heterodimerizes exclusively with β_1_, is important in the maintenance of cell-cell junctions [35]. It is also involved in cell spreading and hemidesmosome stability [35,36]. Depending on the lens type, significant reductions in integrin expression were observed with OFX and ReNu, suggesting that HCEC cell-cell and adhesion to the substrate were being disrupted. This has potential in vivo implications, whereby such a reaction may lead to a disruption in the mechanism of cell adhesion and potentially lead to increased cell shedding. These results are in agreement with a recent in vitro study showing that direct exposure to OFX caused a disruption in the structure of corneal epithelial tight junctions in vitro [37].

The results of this study illustrate the complex system of lens-MPS interactions and the many parameters (lens type/chemistry, buffer, active ingredients, and marker of cytotoxicity) that need to be taken into account when assessing the biocompatibility of MPS with SiHy lenses. From our studies, it is evident that some of the deleterious effect on HCEC viability of OFX and ReNu were due to the buffer used (borate) in the MPS, rather than the biocides themselves. The effect of borate buffer on corneal epithelial cell viability in vitro and in vivo has been discussed before [31,38,39]. With the exception of Lehman et al. [39], our results, which suggest a potential cytotoxic effect of borate buffer, agree with previous reports. The viability, caspase and integrin results from lenses soaked in Complete or SoloCare indicated that the PHMB released from the SiHy lenses was not in a concentration sufficient to induce significant cell damage. Interestingly with ReNu, significant changes in integrin expression were observed and could not be accounted for by a cytotoxic effect of borate buffer. There were also differences between lens type. It could be speculated that the combination of borate and PHMB led to a synergistic cytotoxic effect on integrin expression and that differences between lens types are due to difference in uptake and release of PHMB.

One of the major differences observed with the various lens-solution combinations was with BA-OFX. While the BA-OFX combination led to reduced cell viability and integrin expression similar to the other lenses soaked in OFX, BA-OFX was the only OFX combination that did not cause a significant increase in activated caspases. The amount of OFX release from lenses at 24 h (as determined by UV absorbance) could not explain such a difference in the mechanism of cytotoxicity, as the amount of OFX release by BA-OFX was found to be just as much, if not more, than the other SiHy lenses tested. The difference in cell death mechanisms may be explained more by the release profile of a specific compound such as Aldox (one of the biocides used in OFX) rather than the amount of MPS solution observed at 24 h. Further studies are needed to determine if the difference in mechanism of release as observed by Powell et al. [10] leads to the difference in cell apoptosis, as shown in our experiments with OFX-soaked lenses. A recent study by Wilcox et al. [12] also suggests differences in the mechanism of release between LB and GA, whereby OFX release from GA was faster than LB, which may also explain the difference observed with α_3_ downregulation.

Because some lenses soaked in ReNu and OFX showing similar lower levels of viability and integrin expression, it was hypothesized that the mechanisms of cell death through caspase activation would be similar and may likely be triggered by the presence of borate buffer. However, OFX-soaked lenses (except for BA) led to significant caspase activation, while ReNu-soaked lenses did not. OFR (another MPS containing Polyquad and Aldox but containing a different surfactant system) soaked lenses also led to high caspase activation. Aldox/Polyquad and PHMB have been shown to have very different release profiles from lenses [10,12], which may directly affect cell death mechanism, but the difference in chemistry between the formulations may also contribute to caspase activation. A recent study has shown that, unlike PHMB solutions, after interaction with lenses during the disinfection cycle, OFX retained its bactericidal and fungicidal activity [11]. While it may be ideal to retain this property, the residual active disinfecting agents on lenses may be responsible for inducing caspase activation in cells. The fact that incubation with up to 10% OFX and OFR did not lead to caspase activation further suggests that Polyquad/Aldox interaction with the lens material is responsible for the induction of apoptosis in cells. Further studies are required to characterize how Aldox and Polyquad interactions with lens material may affect structure and cytotoxic properties.

### 

#### Conclusion

The results from this study shows that the effect of MPS-released from a contact lens can be investigated in vitro. As shown by the absence of an effect on cells exposed to a PBS-soaked lens, our results indicate that it is not the presence of the lens that affected cell viability and phenotype, but what was being released from the lens. The in vitro model also demonstrated a lens effect in the mechanism of MPS-induced cell death pathways. The results indicate that OFX-induced cell death may be influenced by the surface properties of certain SiHy lenses. The differences in physical properties of lenses, which affect the uptake and release of the various ingredients in MPS, had a significant effect on caspase activation. Our results demonstrate that solution interactions with SiHy lenses significantly affect cell response and caution should be applied, as mechanisms of activation cannot reliably be predicted from MPS dilution alone.
